# Improving Understanding of and Response to Infodemics During Public Health Emergencies

**DOI:** 10.1089/hs.2021.0044

**Published:** 2021-02-18

**Authors:** Tara Kirk Sell, Divya Hosangadi, Marc Trotochaud, Tina D. Purnat, Tim Nguyen, Sylvie Briand

**Affiliations:** Tara Kirk Sell, PhD, is a Senior Scholar; Divya Hosangadi, MSPH, is a Senior Analyst; and Marc Trotochaud, MSPH, is an Analyst; all at the Johns Hopkins Center for Health Security, Baltimore, MD. Divya Hosangadi and Marc Trotochaud are also Research Associates, and Tara Kirk Sell is also an Assistant Professor; all in the Department of Environmental Health and Engineering, Johns Hopkins Bloomberg School of Public Health, Baltimore, MD. Tina D. Purnat, MS, is a Technical Officer, Digital Health Technologies, Digital Health and Innovation, Science Division; Tim Nguyen, MPH, is Unit Head, High Impact Events Preparedness, and Sylvie Briand, MD, PhD, MPH, is Director; Global Infectious Hazard Preparedness, WHO Emergency Preparedness; all at the World Health Organization, Geneva, Switzerland. The views expressed in this paper are the authors alone and do not represent the views of the authors' respective organizations.

**Keywords:** Infodemic, Misinformation, Risk communication, Public health emergency/response

Effective communication during epidemics and outbreaks is a critical component of a public health response. Even more than usual, people need accurate information so that they can adapt their behavior and protect themselves, their families, and their communities against infection, onward transmission, and death. However, during an epidemic or pandemic, the communication environment can be complicated by an “infodemic,” which is the rapid, large-scale spread of health information and misinformation through a variety of media and informational channels.^1^ This overabundance of information—some accurate and some not—makes it difficult for people to differentiate between false and true information, and has been particularly challenging to address during the ongoing coronavirus disease 2019 (COVID-19) pandemic. Addressing infodemics is a new and centrally important challenge to responding to acute health events. Given its global scale and rapid spread, the current COVID-19 infodemic is an important opportunity to find and adapt new preparedness and response tools to manage the information ecosystem in which we live.

Just as the tactics for health education, social mobilization, and public communication that helped eradicate smallpox over 40 years ago have evolved, so have the tactics used by purveyors of false information. Disinformation has become increasingly sophisticated, hard to track, and emotive and can imperil public trust in health authorities and service delivery.^2,3^ Misinformation and distrust can be a particularly toxic mix that causes people to reject health interventions such as vaccines, as in the case of polio^4^; disregard health guidance, as has been seen with Ebola^5,6^; or try out unproven and dangerous therapies like ingesting methanol to prevent COVID-19.^7^ Disinformation and misinformation can even be used to foment social and political instability,^8^ effectively weaponizing false information.

Information, misinformation, and public health are intertwined by nature. As the world changes, so, too, do emergency response strategies. To organize efforts to manage the infodemic, the World Health Organization (WHO) established a set of 50 proposed actions to tackle the COVID-19 infodemic.^1^ One focus area was to establish a crucially needed public health research agenda. This need led to the organization of the first Global Infodemiology Conference in the summer of 2020. In developing this conference, WHO organizers expanded the conceptualization of infodemiology beyond its traditional definition—the science of distribution and determinants of information in an electronic medium—with an aim to inform public health and public policy. In addition, the organizers incorporated the need to understand the offline–online information ecosystem, the impact of infodemics on health behaviors, and achievement of self-efficacy and resilience to infodemics for both individuals and communities as critical components of infodemiology. The participants discussed the science that informs the management of infodemics, including the systematic use of evidence-based analysis and interventions, and how to mitigate harmful effects of health misinformation on health behaviors during acute health events.

Following the conference, WHO and partners coordinated a joint call for papers with 5 academic journals in different fields, all related to different components of infodemics and infodemic management during public health emergencies. This special feature in *Health Security* is devoted to analysis of health emergency-related infodemics, communication policy needs, and public health practices to overcome misinformation and disinformation during health events. Here, we offer a range of articles focused on different aspects of infodemics and response efforts. Original articles provide practice and research-based analysis of misinformation during epidemics, characteristics of successful online messaging, disinformation and epidemics in the context of biowarfare, the impact of different news sources on risk perception, and the use of community listening and feedback to respond to false information. Commentaries in this special feature focus on the COVID-19 pandemic and crisis and emergency risk communication, scientific situational awareness, and approaches to social media messaging.

The papers included here focus on a wide range of topics related to infodemics and public health emergencies, highlighting the importance of a multifaceted approach to understanding and managing infodemics. Although the existence of false information during public health emergencies will never be completely eradicated, understanding the problem, improving approaches to address it, and a continued commitment to meeting the challenge of infodemics may enable responders and policymakers to lessen its spread and impact.
